# Tonsilolith in Routine Panoramic Radiographies; Is It a Common Incidental Finding?

**DOI:** 10.5812/iranjradiol.7563

**Published:** 2012-06-30

**Authors:** Mohammad Mehdi Aghdasi, Solmaz Valizadeh, Niloofar Amin-Tavakoli, Hooman Bakhshandeh

**Affiliations:** 1 Department of Oral and Maxillofacial Radiology, Faculty of Dentistry, Shahid Beheshti University of Medical Sciences, Tehran, Iran; 2 Department of Epidemiology, Shahid Rajaee Cardiovascular Medical Center, Tehran University of Medical Sciences, Tehran, Iran; 3 Advanced Diagnostic and International Radiology Research Center (ADIR), Tehran University of Medical Sciences, Tehran, Iran

**Keywords:** Radiography, Panoramic, Tonsilolith

Dear Editor

Radiographs which are commonly prescribed may not provide accurate differential diagnosis for various lesions of the head and neck. As a result, higher doses of radiation are imposed to take supplementary radiographs for more precise diagnosis resulting in loss of time and money (1). Tonsiloliths frequently fall into this category. They are calcified masses which form within the crypts of the palatal tonsils and are usually accidentally detected in panoramic radiographs due to their small size and lack of clinical manifestations (2). Diagnosis of larger stones is straight forward due to their clinical signs and symptoms. It is essential that smaller lesions hidden in the depth of the crypts to be diagnosed as they can cause unexplained symptoms and signs; one of which is unexplained halitosis, a symptom that can be a great social hindrance (3, 4, 5, 6).

**Figure 1:**
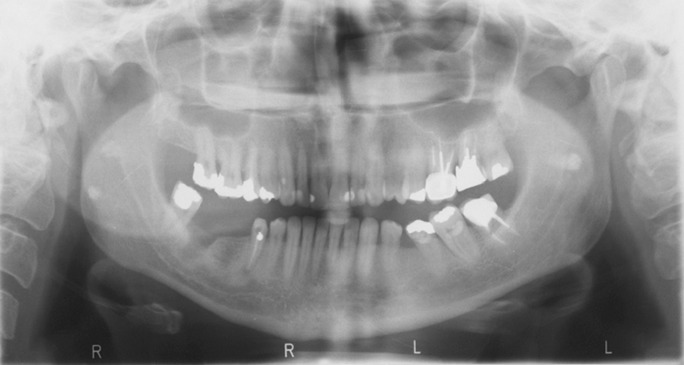
The location, shape and number of tonsiloliths

Tonsiloliths usually appear in the midline of the mandibular ramus on dental panoramic radiographs, where the image of the posterior surface of the tongue crosses the ramus in the palato-glossal or palato-pharyngeal space. Their usual image appears as a cluster of multiple, small radiopacities with ill-defined margins (Figure 1). Despite the fact that such calcifications are seen within a limited area of the image (i.e. the anatomical location of the tonsils) they may be interpreted as other lesions that may cause diagnostic problems and may impose additional radiation and possible unnecessary treatment for the patient (7, 8, 9, 10). In this descriptive study, panoramic radiographs of 966 patients referred to the Department of Radiology from February, 2007 to December, 2008 were assessed in a sequential manner. Three dento-maxillofacial radiologists assessed the radiographs and the final diagnosis of presence or absence of tonsilolith in each case was based on group consensus of all three specialists.

Patients with and without lesions were classified according to their age and gender. Normal distributions and ratios were assessed; Chi square test was carried out to assess the relation between gender and prevalence of tonsilolith and Mann-Whitney U test was used in order to determine probable statistical relation between age and tonsilolith formation. Totally, 470 (48.7%) were men and 496 (51.3%) were women. There were no exclusion criteria for the taken panoramic radiographs. Their mean age was 35.8 ± 1.8 years (range, 7-83). Panoramic radiographs revealed the tonsiloliths in 47 patients. (4.9%). Of these, 29 (61.7%) were men and 18 (38.3%) were women, resulting in a male to female ratio of 1.6/1. Among the remaining patients, 441 (48%) were men and 478 (52%) were women, resulting in a male to female ratio of 0.92/1. In the next step, the frequency of tonsiloliths in different age groups was evaluated. Tonsilolith was more common in older ages. The mean age of patients with tonsilolith was 50 ± 14.1 years, but it was 35 ± 17.9 years in patients without tonsilolith (P < 0.001). The distribution of age groups was considered to be nearly normal. Nearly half of the patients (48.9%) had bilateral tonsiloliths; calcified masses were more frequent on the left side compared to the right (27.7% and 23.4%, respectively). In order to determine the relation between gender and prevalence of tonsiloliths, the P value was 0.067, which indicates that gender, as an independent variable, is not a risk factor for tonsilolith formation. In assessing the possible relation between age and tonsilolith, the P value of less than 0.001 suggested the role of age as a determining factor in tonsilolith formation. Poisson regression model was used to investigate the adjusted associations between age and sex (as predictors) and tonsiloliths. No associations were found after the adjustment; therefore, despite the results of bivariate analysis, neither age nor sex were the predictor of tonsiloliths.

The present study demonstrated that tonsiloliths may be accidentally detected through panoramic radiographs in nearly 5% of cases. There is no gender-based predisposition, and tonsiloliths are more common in the 41 to 60-year-old group.

Tonsiloliths should be the first differential diagnosis when multiple opaque lesions with ill-defined borders superimposed on the palatal uvula and the ramus are detected on a panoramic radiography in a middle-aged patient. A correct diagnosis will eliminate the need for further evaluations including radiography and clinical examinations.
